# On the Role of Reaction Current Distribution to Attain Competitive Solid‐State Batteries

**DOI:** 10.1002/anie.2890151

**Published:** 2026-03-30

**Authors:** Johannes Hartel, Lukas Ketter, Eva Schlautmann, Erik Šimon, Karol Végsö, Poongodi Ayyanusamy, Tim Bernges, Peter Siffalovic, Wolfgang G. Zeier

**Affiliations:** ^1^ Institute of Inorganic and Analytical Chemistry University of Münster Münster Germany; ^2^ International Graduate School of Battery Chemistry Characterization, Analysis, Recycling and Application (BACCARA) University of Münster Münster Germany; ^3^ Centre For Advanced Materials Application Slovak Academy of Sciences Bratislava Slovakia; ^4^ Institute of Materials and Machine Mechanics Slovak Academy of Sciences Bratislava Slovakia; ^5^ Institute of Physics Slovak Academy of Sciences Bratislava Slovakia; ^6^ Forschungszentrum Jülich GmbH Institute of Energy Materials and Devices Helmholtz‐Institute Münster (IMD‐4) Münster Germany

**Keywords:** lithium‐ion battery, *operando* X‐ray diffraction, porous electrode theory, reaction current distribution, state battery

## Abstract

Competitive solid‐state batteries must allow for high areal loadings (> 5 mAh·cm^−2^) and fast charging rates (> 2 C). Nevertheless, current academic research mainly focuses on systems with smaller loadings and lower C‐rates. For established cell chemistries a focus shift is required when aiming toward practical application. Increasing the areal active material content and C‐rates is often accompanied by charge transport limitations in the electrodes. In this work, the role of reaction current distribution in composite electrodes is highlighted as solid‐state batteries advance toward higher areal loadings and charging rates. Using NCM‐argyrodite composites as a case study, we revisit Newman's porous electrode theory in the context of solid‐state batteries to rationalize composite electrode cycling performance. Further, *operando* high‐energy X‐ray diffraction is employed to track lithiation states of NCM across the electrode as a function of state of charge. The results reveal significant improvements in reaction current distribution, when employing faster conducting Li_5.5_PS_4.5_Cl_1.5_ instead of conventional Li_6_PS_5_Cl, underscoring the need for fast lithium‐ion conductors to enable competitive solid‐state batteries. This work demonstrates the importance of precisely controlling electrode composition to balance ionic and electronic transport, ensuring homogeneous utilization of the active material and mitigating local strain and overcharging.

## Graphical abstract

We conducted a case study on reaction current inhomogeneities when increasing areal loadings and charging rates in NCM‐argyrodite composite cathodes for solid‐state batteries. Using a combination of cycling experiments and *operando* high‐energy X‐ray diffraction, we show that employing faster conducting Li_5.5_PS_4.5_Cl_1.5_ instead of Li_6_PS_5_Cl as catholyte yields improved cycling performance as a result of a more homogeneous reaction current distribution.

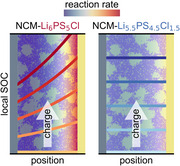

## 1 Introduction

Solid‐state batteries (SSB) are perceived as a promising technology to overcome performance and safety limitations of state‐of‐the‐art lithium‐ion batteries (LIB) [1]. High volumetric energy densities can be achieved by utilizing negative electrodes with high Si content [2], Li metal [3], or Li reservoir‐free negative electrodes [4] together with high‐capacity positive electrodes incorporating, for example, Ni‐rich layered oxides (NCM, LiNi*_x_* Co*_y_* Mn*_z_* O_2_) [5]. At the same time, composite electrodes need to employ electrochemically stable solid electrolytes (SE) with ionic conductivities of about 10 mS·cm^−1^ (e.g., specific lithium argyrodites [6] or oxyhalides [7]) to enable high power densities [8, 9]. To be a competitive battery technology, SSB need to allow C‐rates of ≥  2 C and areal loadings of ≥  5 mAh·cm^−2^. Current academic SSB research, however, often considers cells with active material loadings < 2 mAh·cm^−2^ and C‐rates < 1 C [10].

This approach is valid for fundamental investigations and “proof‐of‐concept” studies. Nevertheless, research on more established electrode chemistries, including NCM‐Li_6_PS_5_Cl composite positive electrodes requires a shift in focus toward more realistic active material loadings and current densities to reliably evaluate competitiveness with current LIB. Otherwise, cycling data could suggest high capacities and Coulomb efficiencies that cannot be realized in practical SSB.

**FIGURE 1:**
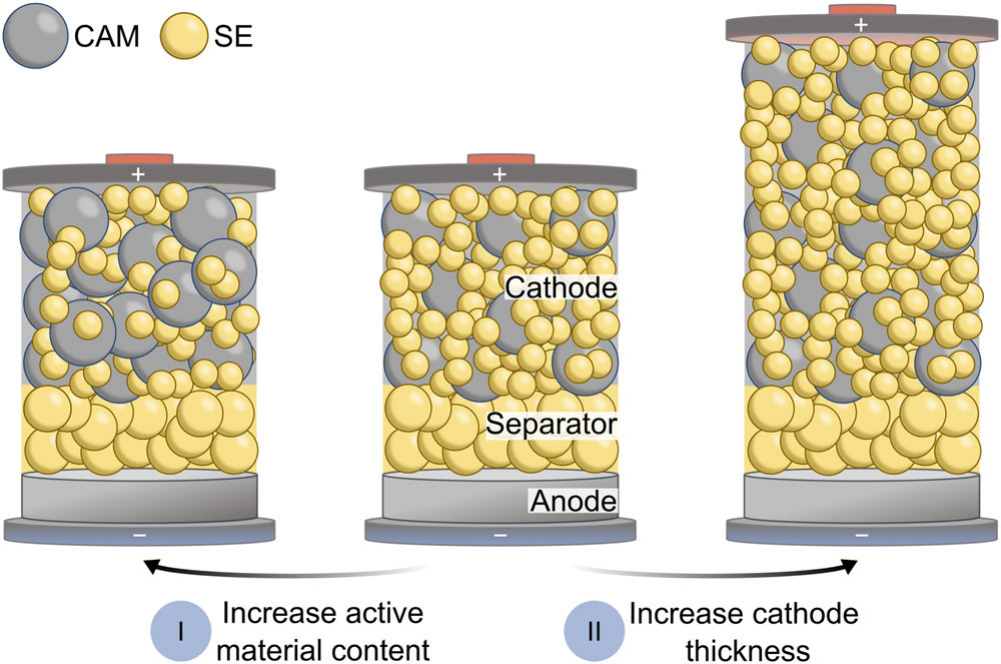
Approaches to enhance areal loading of composite electrodes. Schematic showing possibilities to increase areal loadings by (I) utilizing higher active material contents and (II) increasing electrode thickness (exemplarily shown for a composite cathode).

To enhance energy densities in SSB, composite electrodes can be optimized accordingly by (I) increasing the active material content and (II) by increasing the thickness of the composite electrodes (Figure 1). While the former method is desirable, as it reduces the amount of electrochemically inactive SE, it is strongly limited by loss of ionic percolation [11]. These ionic transport limitations can result in incomplete active material utilization and nonuniform reaction distributions, especially at high C‐rates [12]. Through *operando* neutron analysis, Bradbury et al. observed electrode reactions to occur almost exclusively near the interface of separator and composite cathode at the beginning of discharge. With progressing discharge, the electrochemical reaction front propagates toward the current collector [13]. Lithiation gradients as a result of inhomogeneous reaction current distribution have also been revealed in high‐loading NCM‐Li_6_PS_5_Cl composite cathodes by Stavola et al. [14]. Utilizing energy‐dispersive X‐ray diffraction, the researchers revealed faster reaction rates near the separator in electrodes with 80 wt.% NCM, whereas for electrodes with 40 wt.% NCM faster reaction rates were observed near the current collector. Davis et al. demonstrated how electrode composition affects the reaction current distribution [15]. Using graphite‐Li_6_PS_5_Cl composites as a model system, the researchers found highly inhomogeneous lithiation behavior for electrodes with high active material content. Increasing the Li_6_PS_5_Cl fraction, however, leads to homogeneous lithiation behavior. The heterogeneity of the reaction rate distribution was found to clearly influence the rate capabilities of the various electrodes investigated. To mitigate charge transport limitations and increase active material utilization, major efforts have been made to optimize the electrode architecture, for example, by varying the particle size distribution of the SE [16] and active material [17], by gradient electrode design [18, 19], or by introducing faster conducting SE [20]. Nevertheless, to realize the targeted areal loadings and energy densities, increasing the electrode thickness beyond typical values used in academic research (≈  60 µm) is inevitable. This change also comes with further challenges, including the amplification of transport‐related performance issues as well as additional manufacturing challenges [21]. Considering that commercial SSB will need to be low‐pressure pouch cells, these limitations will be even more severe due to their increased porosity [22, 23].

In this context, we identify reaction current inhomogeneities as a bottleneck, since key metrics required for advancing toward high areal loadings and current densities inherently exacerbate these inhomogeneities, ultimately limiting battery performance. We start by revisiting porous electrode theory in the context of SSB, providing a general framework for understanding the origin of heterogeneous reaction current distributions. Building on this, we then turn to a practical case study illustrating the importance of reaction current inhomogeneities when increasing areal loadings and charging rates using NCM‐argyrodite composite cathodes. Using a combination of cycling‐ and *operando* high‐energy X‐ray diffraction (HEXRD) experiments, we show that employing faster conducting Li_5.5_PS_4.5_Cl_1.5_ instead of Li_6_PS_5_Cl as catholyte yields improved cycling performance as a result of a more homogeneous reaction current distribution during operation.

## 2 Results and Discussion

### 2.1 Porous Electrode Theory in the Context of Solid‐State Batteries

**FIGURE 2:**
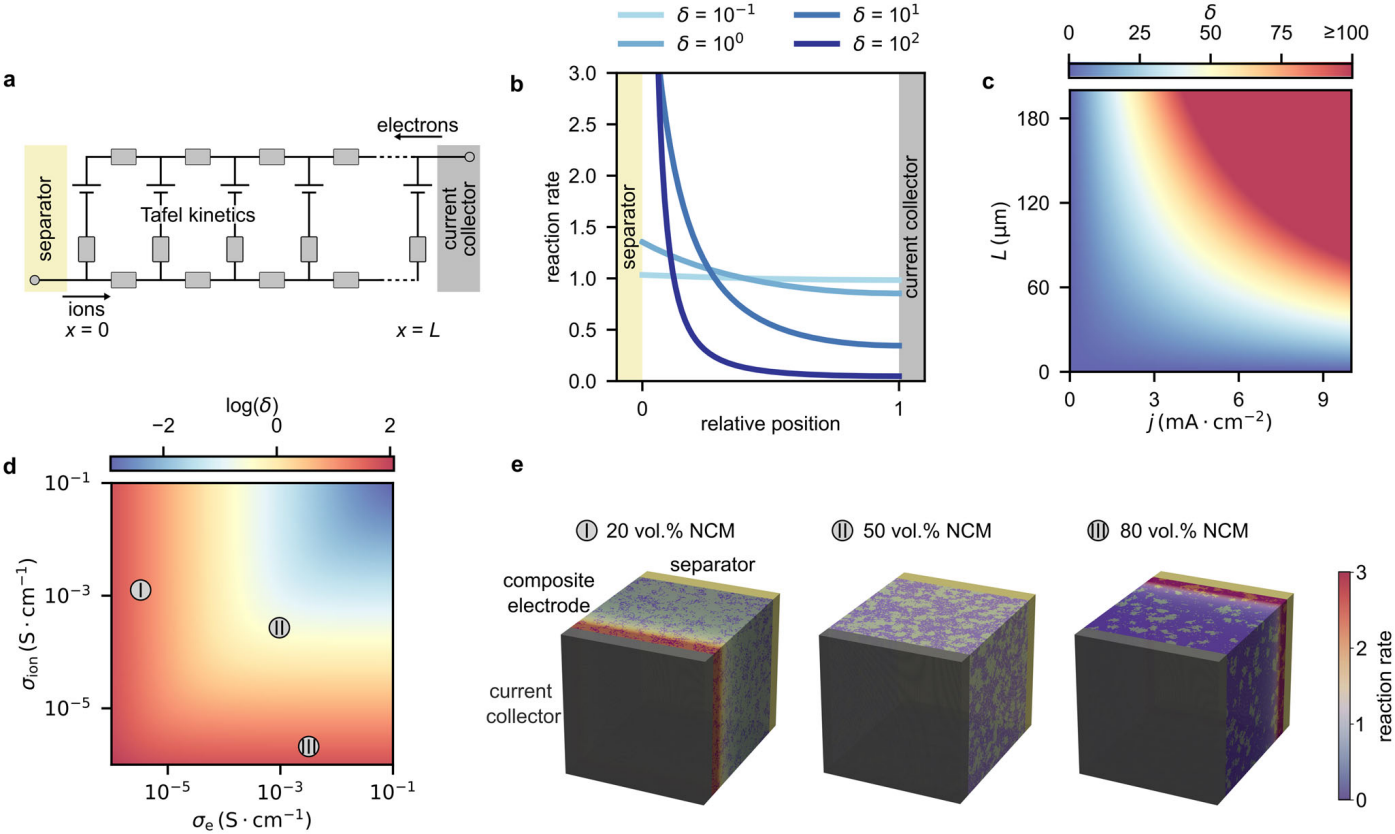
Applying porous electrode theory to solid‐state battery electrodes. For *δ*‐parameter calculations on the example of NCM‐Argyrodite composites, *T *= 298.15 K and *n*_e_ = 1 were assumed. (a) Equivalent circuit representing a porous electrode according to Newman et al. [27]. (b) Reaction rate as a function of relative position within the porous electrode for varying *δ* schematically illustrated for the case where *σ*_ion_ ≪  *σ*_e_ (adopted from Newman and Tobias) [25]. (c) *δ*‐parameter for varying electrode length and current densities of a NCM83‐Li_5.5_PS_4.5_Cl_1.5_ electrode with 80 wt.% NCM measured in this work (*σ*_ion _= 0.016 mS·cm^−1^ and *σ*_e _= 0.52 mS·cm^−1^). (d) *δ*‐parameter for varying partial electronic and ionic conductivities with *j* = 0.5 mA·cm^−2^ and *L* = 60 µm. (e) Reaction rate distribution in virtual composites representing an electrode of 60 µm and consisting of different volume fractions of active material (NCM) and SE (Li_6_PS_5_Cl). *σ*_ion,SE _= 2.33 mS·cm^−1^ and *σ*_e,NCM _= 5.22 mS·cm^−1^ have been assumed to calculate effective ionic and electronic composite conductivities [11]. Further a current density of 0.5 mA·cm^−2^ has been assumed for the calculations of the respective reaction rate distributions.

Although originally developed for porous active material filled with liquid electrolyte, Newman's porous electrode theory can be applied to composite electrodes in SSB, as the general considerations remain the same [13, 24]. Important differences in SSB regarding porous electrode theory are outlined in section S1.1. The electrode is assumed to be a homogeneous component of length *L* located between the electrolyte solution, or in the case of SSB a SE separator, at position *x *= 0 and the current collector at *x = L* [25, 26]. The electrical equivalent representation of the theoretical one‐dimensional electrode is shown in Figure 2a and consists of two interconnected branches [27]. While one of the branches represents purely ionic charge transport through the electrolyte phase, the other reflects exclusively electronic transport through the active material. The ionic (*σ*_ion_) and electronic conductivity (*σ*_e_) of the two branches correspond to the effective ionic and electronic conductivity of the electrode and link the potential gradient in the electrolyte phase $\left(\frac{d\phi_{\mathrm{El}}}{dx}\right)$ and the active material $\left(\frac{d\phi_{\mathrm{AM}}}{dx}\right)$ to the ionic (*i*_ion_) and electronic flux density (*i*_e_) going through the electrode respectively via Ohm's law (Equation 1) [28].

(1) $$\;i_{e}=-\sigma_{e}\frac{d\phi_{\mathrm{AM}}}{dx}\;\mathrm{and}\;i_{\mathrm{ion}}=-\sigma_{\mathrm{ion}}\frac{d\phi_{\mathrm{El}}}{dx}$$

The interconnections between the two branches in the electrical equivalent picture (Figure 2a) model electrode reactions taking place at interfaces between electrolyte and active material. Changes in ionic and electronic flux density with position along the branches are caused by these electrode reactions and are proportional to the specific interface area (*a*) and the reaction rate, which is a function of potential difference between active material and electrolyte $f(\phi_{\mathrm{AM}}-\phi_{\mathrm{EL}})$. Due to current conservation, the changes in ionic and electronic flux densities are correlated (Equation 2) [25, 26].

(2) $$\;\frac{di_{e}}{dx}=\frac{-di_{\mathrm{ion}}}{dx}=af\left(\phi_{\mathrm{AM}}-\phi_{\mathrm{EL}}\right)$$

Newman and Tobias demonstrated that the above considerations can be combined in a differential equation when assuming Tafel polarization behavior for charge transfer. Choosing boundary conditions where the total current density (*I*) is carried exclusively by electrons at the current collector interface (*x = L*) and solely by ions at the electrolyte interface (*x = *0), and by defining $\phi_{\mathrm{El},\;x=0}=0$ as an arbitrary reference potential, they were able to derive an analytical solution for the dimensionless reaction rate $\frac{dj}{dy}$ with $j=\frac{i_{e}}{I}$ and $y=\frac{x}{L}$ being the relative current density and relative position (details in section S1.2). The solution contains the dimensionless parameter δ which determines the uniformity of the reaction rate distribution in the electrode at the instance of closing the circuit (Equation S1). For symmetric charge transfer coefficients in the polarization equation, *δ* is defined as:

(3) $$\delta =L\left|I\right|\frac{0.5n_{e}F}{RT}\left(\frac{1}{\sigma_{e}}+\frac{1}{\sigma_{\mathrm{ion}}}\right)$$

where *n*_e_ represents the number of electrons transferred per electrode reaction [25, 26].

To emphasize their relationship, reaction rate distributions for *δ* parameters of varying orders of magnitude are discussed in the following. Figure 2b shows such distributions for the case when ionic transport is assumed to be much more sluggish than the transport of electrons (*σ*_ion_ ≪  *σ*_e_). As the current tends to follow the path of least resistance through the electrode, higher reaction rates are predicted near the interface between the separator and the composite electrode (further cases are discussed in section S1.3). The nonuniformity of the reaction rate distribution within the electrode increases drastically with the order of magnitude of *δ* [25].

From a practical perspective these reaction inhomogeneities cause a multitude of problems. First, only incomplete utilization of the active material is achieved across the electrode. Second, localized current hot spots and thereby overcharging and ultimately accelerated degradation can be expected [29]. While the *δ*‐parameter captures the reaction current distribution only at the moment of closing the circuit, in practice, the distribution evolves with the state of charge (SOC) or state of discharge (SOD) of the electrode. This will affect local partial conductivities and leads to spatial variations in the concentration of the electrochemically active species. Nevertheless, experimental studies by Davis et al. and Stavola et al. have shown that composite electrodes exhibiting significant initial inhomogeneities in reaction current distribution will continue to show inhomogeneities in reaction current distribution throughout cycling [14, 15]. This observation is also supported by chemical intuition, as regions of local over(dis‐)charging driven by reaction current inhomogeneities are likely to exacerbate degradation, particularly in systems with high *δ*. Considering that *δ* and the reaction rate depend on electrode thickness and current density, these detrimental effects can be expected for realistic SSB configurations. In Figure 2c, the dependence of *δ* on the electrode length and current density is shown, assuming *n*_e_ = 1 and effective conductivities of a LiNi_0.83_Co_0.11_Mn_0.06_O_2_ (NCM83)‐Li_5.5_PS_4.5_Cl_1.5_ composite (80:20 weight ratio) measured in this work (Figure S4). While low *δ‐*parameters and thus homogeneous electrode reactions are expected when thin electrodes and low current densities are used, limitations regarding nonuniform reaction rates are quickly reached when increasing the electrode thickness and current density. By tailoring the electrode composition in terms of effective conductivities, *δ* can be influenced drastically (detailed discussion in section S1.4) [24]. Figure 2d highlights this dependence of *δ* on the effective conductivities, assuming *n*_e_ = 1, a current density of 0.5 mA·cm^−2^ and a typical academic research electrode thickness of 60 µm. To further emphasize these effects, the effective ionic and electronic conductivities of three virtual NCM83‐Li_6_PS_5_Cl composites with NCM volume fractions of 20%, 50%, and 80% have been calculated using a resistor network model, described previously (Figure 2e) [11]. While a small *δ‐*parameter and thus a homogeneous reaction rate distribution is obtained in the case of equal volume fractions, high *δ*‐parameters and inhomogeneous reaction rates are observed when moving toward high volume fractions of either NCM83 or Li_6_PS_5_Cl. For composites with electronic transport limitations, high reaction rates are expected close to the current collector, while conversely, for ionic transport limitations, high reaction rates are expected close to the separator (Figure S1). This highlights the importance of effective transport optimization in composite electrodes to prevent inhomogeneous reactions rates and thus performance limitations.

### 2.2 Toward High Active Material Loadings

To demonstrate the particular relevance of reaction current distribution when shifting toward high areal loadings and charging rates, cycling experiments on high‐loading NCM‐argyrodite cathode composites were performed, and spatially‐scanning HEXRD was conducted to investigate reaction current distribution at different SOC. The experimental details can be found in section S2. Specifically, NCM83‐Li_6_PS_5_Cl and NCM83‐Li_5.5_PS_4.5_Cl_1.5_ composite cathodes with CAM loadings of 5, 6, and 7 mAh∙cm^−2^ and a CAM to SE mass ratio of 80:20 were selected as a model system, since the SE are expected to exhibit different ionic conductivities while having similar properties otherwise. Ionic conductivities of 2.5(1) and 7.8(1) mS∙cm^−1^ were measured for Li_6_PS_5_Cl and Li_5.5_PS_4.5_Cl_1.5_, respectively, in agreement with literature reports (Figure S3b) [6, 30]. Furthermore, their particle size was found to be comparable with D50_vol_ of approximately 13 µm (Figure S3c). Therefore, only a minor influence of microstructural differences on the half‐cell performance is expected. As both SE belong to the argyrodite structural family and contain similar elemental constituents, their (electro)chemical stability and mechanical properties can be considered reasonably comparable and it is fair to expect that these do not significantly influence their performance as catholyte in composite cathodes [31, 32].

**FIGURE 3:**
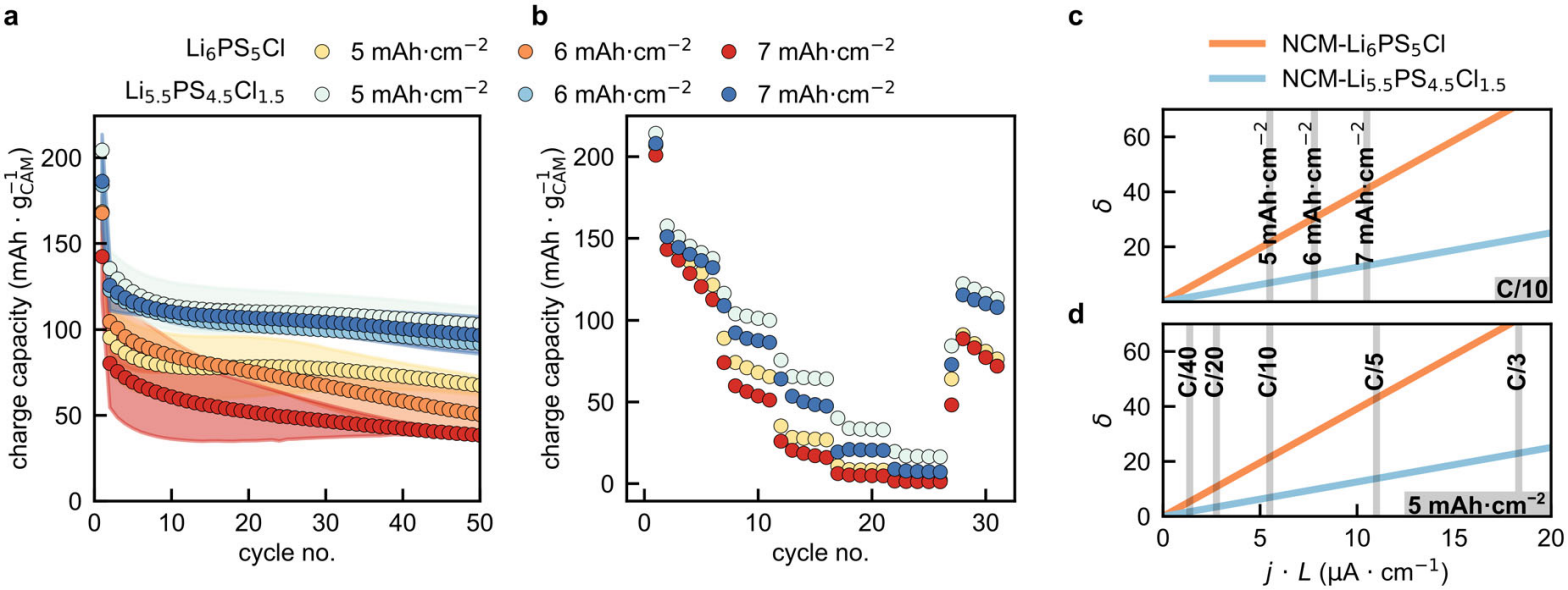
Relevance of reaction current distribution for high‐loading composite cathodes. (a) Comparison of the charge capacities at a rate of C/10 using Li_6_PS_5_Cl and Li_5.5_PS_4.5_Cl_1.5_ catholytes with different areal NCM83 loadings. (b) Comparison of the charge capacities for different C‐rates, areal loadings and catholytes. (c) *δ* as a function of *j·L* at a fixed rate of C/10 and different active material loadings (d) *δ* as a function of *j∙L* at a fixed areal loading of 5 mA·cm^−2^ and different C‐rates.

Charge capacities from half‐cell cycling experiments performed at C/10 are shown as a function of cycle number in Figure 3a. The data reveal consistently superior performance when utilizing the faster Li_5.5_PS_4.5_Cl_1.5_ catholyte. Interestingly, while the half‐cells with NCM83‐Li_5.5_PS_4.5_Cl_1.5_ provide similar charge capacities irrespective of the active material loading, a clear trend of lower charge capacities for thicker electrodes arises when NCM83‐Li_6_PS_5_Cl composites are employed as positive electrodes.

To corroborate the trends, *δ‐*parameters (Equation 3) are calculated based on the experimental effective conductivities *σ*_e_ and *σ*_ion_ (Figure S4) and *n*_e_ = 1 for each positive electrode configuration and are shown in Figure 3c as a function of *j* ∙ *L*. The intersections with the gray vertical lines mark the *δ‐*parameters of the respective composite electrodes with approximately 110, 130, and 150 µm thickness cycled at C/10, corresponding to the areal loadings of 5, 6, and 7 mAh·cm^−2^. Comparing the *δ‐*parameters of NCM83‐Li_6_PS_5_Cl and NCM83‐Li_5.5_PS_4.5_Cl_1.5_ composites, the Li_6_PS_5_Cl containing composites show significantly larger values across all investigated active material loadings, indicating more inhomogeneous reaction current distributions. This correlates well with the observed higher charge capacities of half‐cells employing NCM83‐Li_5.5_PS_4.5_Cl_1.5_ composite electrodes. In contrast, the more homogeneous initial reaction current distributions indicated by the comparably lower *δ‐*values of the NCM83‐Li_5.5_PS_4.5_Cl_1.5_ composite cathodes, result in superior cycling behavior. Performance limitations at high areal loadings are expected to become more pronounced at low temperatures, as temperature directly affects the reaction current distribution and reduces the partial conductivities according to the activation energies *E*_a_ of the SE (see Equation 3) [33].

Additionally, C‐rate tests were carried out in half‐cells with a CAM loading of 5 and 7 mAh·cm^−2^ (Figure 3b). Analogous to the C/10 cycling experiments, the trends of the charge capacities are in accordance with changes of the *δ‐*parameter. Both, the higher partial ionic conductivities of NCM83‐Li_5.5_PS_4.5_Cl_1.5_ composites, and lower *L* and *j* for positive electrodes with a CAM loading of 5 mAh·cm^−2^, lead to higher charge capacities. The differences in charge capacity and *δ‐*parameter between CAM loadings and catholytes are marginal at low current densities (C/40) and become increasingly pronounced at higher current densities (Figure 3d).

One should note the stronger capacity fading in the C‐rate test compared to the cycling experiments conducted at C/10. This is likely due to prolonged exposure of the argyrodite electrolytes to potentials ≥ 3.5 V versus In/InLi at the low C‐rates of C/40 and C/20. At these elevated potentials, the argyrodite solid electrolyte and NCM83 are known to undergo (electro‐)chemical decomposition reactions leading to the formation of resistive interphases [31, 34]. While the general trends are still captured by the *δ*‐parameter, it does not include decomposition effects, which can complicate qualitative comparisons in cases where decomposition of materials strongly differs.

**FIGURE 4:**
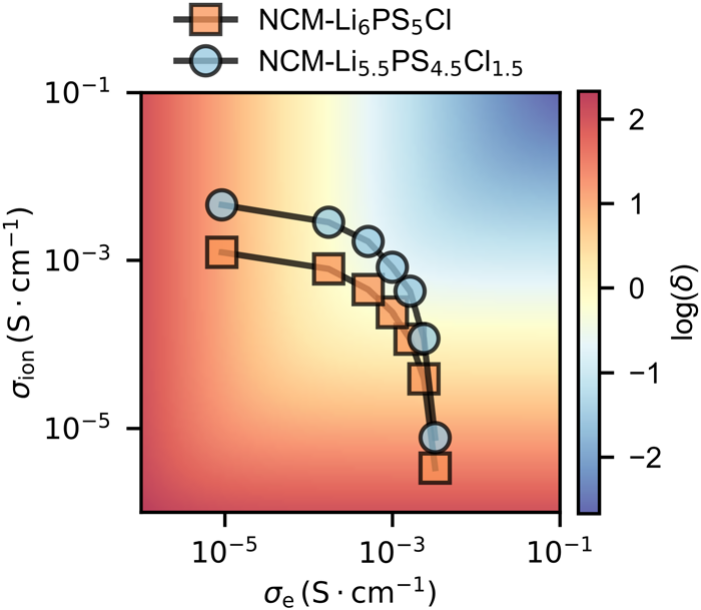
Effective conductivities of NCM composites with Li_6_PS_5_Cl and Li_5.5_PS_4.5_Cl_1.5_ calculated using a resistor network model. The conductivities are mapped to corresponding *δ*‐parameters assuming *L* = 110 µm and *j* = 0.5 mA·cm^−2^.

To highlight the general implications of using SE with higher ionic conductivity in composite electrodes, the partial conductivities for various NCM83 to SE ratios were simulated using a resistor network model and mapped to corresponding *δ*‐parameters at a given length of 110 µm and a current density of 0.5 mA·cm^−2^ on a heatmap (Figure 4), showing the *δ‐*parameter for each combination of *σ*_e_ and *σ*_ion_. When employing the faster‐conducting Li_5.5_PS_4.5_Cl_1.5_, higher partial ionic conductivities appear for all NCM83 to SE ratios relative to Li_6_PS_5_Cl, leading to consistently lower *δ‐*parameters. Hence, when substituting Li_5.5_PS_4.5_Cl_1.5_ with NCM to increase the active material loading in a composite, *δ* remains lower compared to the corresponding NCM‐Li_6_PS_5_Cl composite, thereby mitigating the occurrence of reaction fronts. To maintain sufficient electronic connection between the electrode active material particles, conducting carbons are typically added [35]. However, to compensate for the reduced ionic conductivity faster ionic conductors will be needed.

**FIGURE 5:**
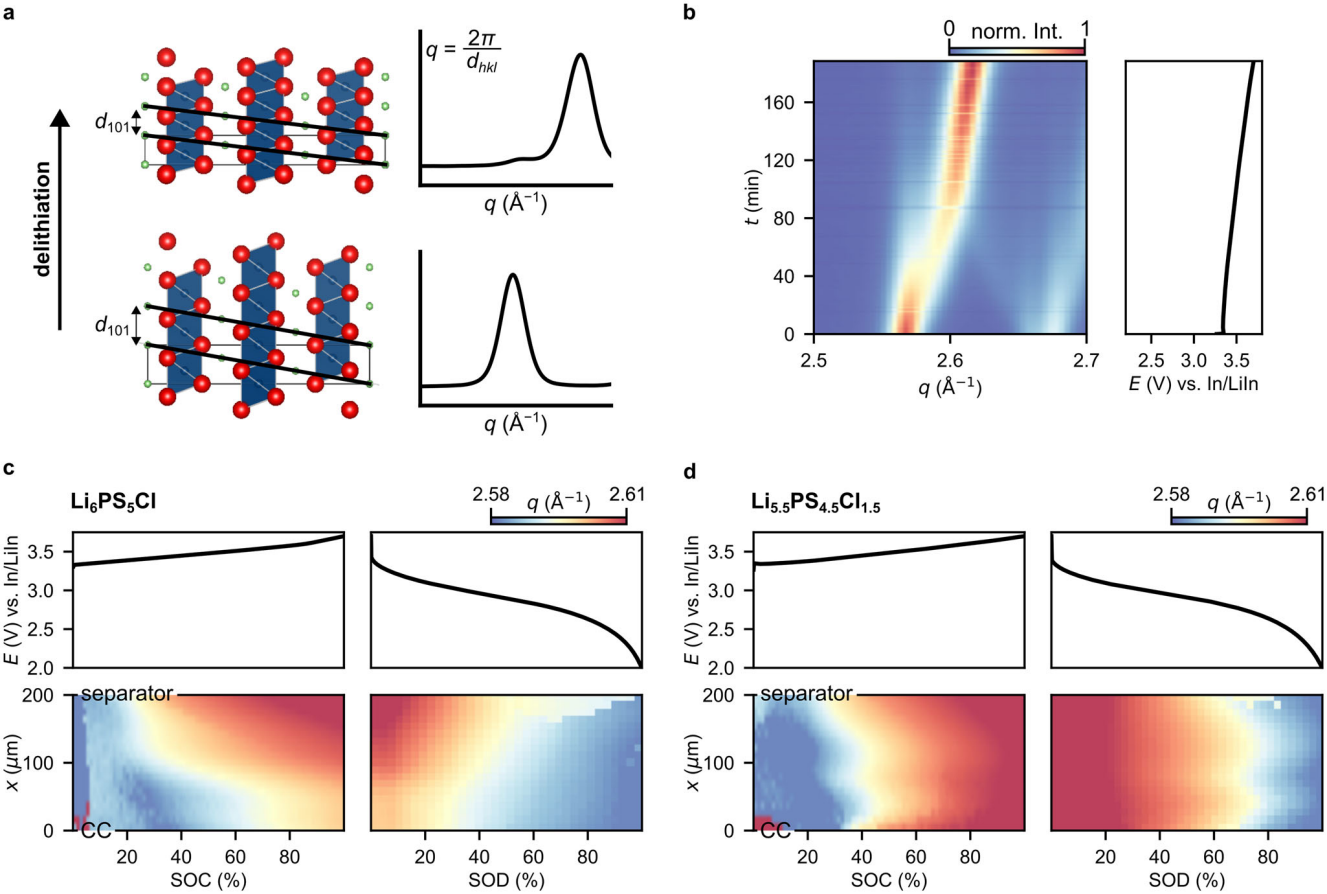
*Operando* HEXRD. (a) Displacement of (101) NCM83 reflection during charge with (b) changes in the NCM83 lattice and the associated changes in the diffraction pattern. The reflection shifts to a higher *q* value due to a decrease in the *a*‐lattice parameter. Voltage change and displacement of (101) reflection according to SOC and discharge of cathode composites with (c) Li_6_PS_5_Cl and (d) Li_5.5_PS_4.5_Cl_1.5_. Displacement of (101) reflection shows more uniform delithiation and lithiation in the cathode with Li_5.5_PS_4.5_Cl_1.5_.

To visualize differences in reaction current, *operando* HEXRD was performed. Specifically, the highest loading used in the cycling experiments of 7 mAh·cm^−2^ was employed as the differences are expected to be most severe based on the cycling performance and the calculated *δ* values. Therefore, the positive electrode of a NCM83‐argyrodite ǀ Li_5.5_PS_4.5_Cl_1.5_ ǀ In/LiIn cell was scanned with *operando* HEXRD from the current collector to the separator. The cells were built in a custom‐made cell setup which was previously validated [36]. The cell setup, assembly procedure, and a scan with the collimated X‐ray beam of the complete cell stack are shown in Figure S5. Based on shifts of the reflections, the lithiation of NCM83 can be determined. Therefore, the (101) reflection of NCM83 was selected to monitor the degree of lithiation as it combines high intensity with a significant shift upon (de‐)lithiation. During delithiation, the lattice parameter *a* decreases, causing a reflection shift toward higher *q*‐values (Figure 5a,b). Oxidation of transition metals reduces their ionic radii and thus the TM–TM distance [37]. During the first delithiation, the (101) reflection is splitting into two reflections due to an irreversible phase transition from the H1 to the H2 phase (Figure S6) [38, 39]. As the H1 phase vanishes, only the (101) reflection of the H2 phase is investigated. SOC and SOD refer to the achieved capacity during the respective charge or discharge process. While the normalized data does not allow for any conclusions regarding Coulomb efficiency, it allows for direct comparison of the effects of different SE in terms of reaction distribution. Cycling data and further results versus capacity are included in the Figure S6.

Figure 5c shows a (101) reflection shift to a higher *q*‐value due to the delithiation of NCM83 in the cathode composite with Li_6_PS_5_Cl. The (101) reflection displacement begins at 20% SOC, and it is starting close to the separator (*x* = 100–200 µm). In contrast, delithiation of the NCM83 close to the current collector (*x* = 0–100 µm) only occurs at SOC above 50% as evidenced by the delayed shift of the (101) reflection. The distribution of the displacement indicates a delithiation gradient with an increased delithiation on the separator side during charge. This is consistent with the findings of Stavola et al., who also observed preferential delithiation close to the separator when using high NCM content in cathodes with Li_6_PS_5_Cl [14]. During discharge, the (101) reflection is shifting back to lower *q‐*values. The lithiation gradient across the cathode with Li_6_PS_5_Cl remains visible up to 75% SOD. At the end of discharge, the (101) reflection is shifted back to the starting position of 2.58 Å^−1^ over the complete cathode length, indicating a reversible lithiation and elimination of the lithiation gradient. Figure 5d shows the charge and discharge processes of the NCM83 cathode with Li_5.5_PS_4.5_Cl_1.5_. The displacement of the (101) reflection starts at 20% SOC. The displacement and therefore the delithiation starts close to the separator, which is consistent with the electrode with Li_6_PS_5_Cl. The displacement of the reflection for the complete cathode is visible at 40% SOC. A small delithiation gradient can still be seen. However, this gradient is significantly less pronounced than in the electrode with the lower ionically conducting Li_6_PS_5_Cl. At the end of the charge, delithiation occurred across the complete cathode length, as indicated by the complete shift of the (101) reflection. The (101) reflection shifts back to a lower *q* value during discharge. The distribution of the displacement is uniform across the thickness of the cathode. This indicates a more homogeneous lithiation of the electrode with Li_5.5_PS_4.5_Cl_1.5_ during the discharge process. To further link the heterogeneous reaction distributions to chemomechanical degradation, strain analyses are performed and discussed in the Section S4.2.

The comparison of the shifts in the (101) reflection of the *operando* HEXRD from the electrodes with Li_6_PS_5_Cl and Li_5.5_PS_4.5_Cl_1.5_ highlights a more homogeneous delithiation and lithiation of the electrode with Li_5.5_PS_4.5_Cl_1.5_ at an areal loading of 7 mAh·cm^−2^. Employing Li_5.5_PS_4.5_Cl_1.5_ with higher ionic bulk conductivity, the effective ionic conductivity of the composite electrode is increased. This is shown in partial transport measurements of the pristine composite electrodes (Figure S4). This is in line with the results of the cycling experiments presented in Figure 3 and the calculated *δ* based on porous electrode theory by Newman and Tobias [25, 26], which predict a more uniform reaction distribution when using Li_5.5_PS_4.5_Cl_1.5_. The reaction distribution across the thickness of the cathode with Li_5.5_PS_4.5_Cl_1.5_ is comparatively more uniform. These results demonstrate that fast lithium‐ion conductors are essential for good electrochemical performance of composite electrodes, especially when aiming for a high active material content and a high loading cathode.

## 3 Conclusion

Heterogeneous reaction current distributions constitute a major limitation for achieving high‐performance SSB, particularly at elevated areal loadings and charging rates required for competitive SSB. Using porous electrode theory as a simple theoretical framework, we show key parameters governing the reaction current distribution. In a case study employing high‐loading NCM‐argyrodite composites we investigate cycling performance and local lithiation states of NCM83 during operation. We demonstrate how employing the faster ion conductor Li_5.5_PS_4.5_Cl_1.5_ leads to more uniform reaction current distributions during operation compared to composites employing Li_6_PS_5_Cl, ultimately supporting improved cycling performance.

Discussing the performance trends in NCM‐argyrodite composite cathodes in the context of reaction rate inhomogeneities underscores the need for precise optimization of electrode composition to balance ionic and electronic transport. Material volume fractions must be re‐optimized when modifying the electrode (e.g., introducing different materials or changing their particle size), as imbalances can cause reaction fronts. Overall, understanding reaction rate distributions is essential to mitigate detrimental follow‐up effects such as local strain evolution and localized overcharging, which accelerate mechanical and electrochemical degradation.

## Conflicts of Interest

The authors declare no conflicts of interest.

## Supporting information



The authors have cited additional references within the Supporting Information [1–18]. **Supporting File 1**: anie71993‐sup‐0001‐SuppMat.pdf.

## Data Availability

The data that support the findings of this study are openly available at http://doi.org/10.17879/70938703822.
