# Supplementary material for: On the Role of Reaction Current Distribution to Attain Competitive Solid‐State Batteries
Source: Angew Chem Int Ed Engl. 2026 Mar 30;65(20):e2890151. doi: 10.1002/anie.2890151 (PMC13159411; doi:10.1002/anie.2890151)
Supplement: Supplementary file 1 — The authors have cited additional references within the Supporting Information [1–18]. Supporting File 1: anie71993‐sup‐0001‐SuppMat.pdf. [file ANIE-65-e2890151-s001.pdf]

Supporting Information - On the Role of Reaction Current Distribution to Attain Competitive Solid-State Batteries

Johannes Hartel§ [a], Lukas Ketter§ [a,b], Eva Schlautmann§ [a], Erik Šimon [c,d], Karol Végsö [c,e], Poongodi Ayyanusamy [e], Tim Bernges [a], Peter Siffalovic* [c,e], , Wolfgang G. Zeier* [a,b,f]

Dedication

* Corresponding author

§ These authors contributed equally

[a] J. Hartel, L. Ketter, E. Schlautmann, Dr. T. Bernges, Prof. W. G. Zeier
Institute of Inorganic and Analytical Chemistry
University of Münster
Corrensstr. 28/30, 48149 Münster, Germany
Email: wzeier@uni-muenster.de

[b] L. Ketter, Prof. W.G. Zeier
International Graduate School of Battery Chemistry, Characterization, Analysis, Recycling and Application (BACCARA)
University of Münster
Corrensstraße 40, 48149 Münster, Germany
Email: wzeier@uni-muenster.de

[c] Dr. E. Šimon, Dr. K. Végsö, Prof. P. Siffalovic
Centre for Advanced Materials Application
Slovak Academy of Sciences
Dúbravská cesta 9, 84513 Bratislava, Slovakia
Email: peter.siffalovic@savba.sk

[d] Dr. E. Šimon
Institute of Materials and Machine Mechanics
Slovak Academy of Sciences
Dúbravská cesta 9, 84513 Bratislava, Slovakia

[e] P. Ayyanusamy, Dr. K. Végsö, Prof. P. Siffalovic
Institute of Physics
Slovak Academy of Sciences
Dúbravská cesta 9, 84513 Bratislava, Slovakia
Email: peter.siffalovic@savba.sk

[f] Prof. W.G. Zeier
Forschungszentrum Jülich GmbH, Institute of Energy Materials and Devices
Helmholtz-Institute Münster (IMD-4)
Corrensstraße 40, 48149 Münster, Germany
Email: wzeier@uni-muenster.de

S1 Supporting discussion on porous electrode theory

1.1 Considerations when applying Newman's porous electrode theory to solid-state batteries

A fundamental distinction between solid and liquid electrolytes lies in the lithium-ion density per unit volume required to attain high ionic conductivities of ≈ 10 mS·cm^{-1}. In solid-state systems, the inherently lower Li^+ mobility must be offset by a significantly higher Li^+ density. For sulfide-based solid electrolytes, the Li^+ density is up to two orders of magnitude greater than that in typical liquid electrolytes.[1] As a result, Li^+ concentrations in solid electrolytes are substantially higher, which means that the relative change in charge carrier concentration (expressed as $\xi = \frac{c_1}{c_1^0}$) will not be severely affected during battery operation. In their work, Newman and Tobias argue that local concentration variations in Li^+ concentration within liquid electrolytes are the primary driver of changes in reaction current distribution during (dis-)charge. While this holds for lithium-ion batteries with liquid electrolytes, it cannot be directly applied to solid-state batteries, where the high baseline Li^+ density renders the concentration of Li^+ comparatively stable during operation.

1.2: Dimensionless reaction rates according to Newman and Tobias

Using the assumptions described in the manuscript, Newman and Tobias were able to derive an analytical expression for the dimensionless reaction rate $\left(\frac{\mathrm{d}j}{\mathrm{d}y}\right)$ as a function of relative position (y) in the electrode (Eq. S1).[2]

$$\frac{dj}{dy} = \frac{2\theta^2}{\delta}\sec^2(\theta y - \psi) \qquad \text{(S1)}$$

To calculate $\frac{dj}{dy}$, besides $\delta = L|I|\beta\left(\frac{1}{\sigma_\mathrm{e}} + \frac{1}{\sigma_\mathrm{ion}}\right)$, the integration constants θ and ψ must be known. For θ and ψ, the relationships Eq. S2 and Eq. S3 apply, where $\epsilon = L|I|\beta\frac{1}{\sigma_\mathrm{ion}}$.

$$\tan(\theta) - \frac{2\delta\theta}{4\theta^2 - \varepsilon(\delta - \epsilon)} = 0 \text{ for } 0 < \theta < \pi \qquad \text{(S2)}$$

$$\psi = \tan^{-1}\left(\frac{\epsilon}{2\theta}\right) \qquad \text{(S3)}$$

By solving Eq. S2 numerically with θ as a variable, θ can be determined. This value can then be used in Eq. S3 to also calculate ψ. After determining the integration constants, Eq. S1 can be utilized to calculate reaction rate distributions.[2]

1.3 Reaction rate distributions

Besides δ, which controls the nonuniformity, also the ratio of ϵ to δ governs the overall shape of the reaction rate distribution. The ratio of ϵ to δ is controlled by the relation of σ_e and σ_ion and determines the side on which electrode reactions predominantly take place.[2]

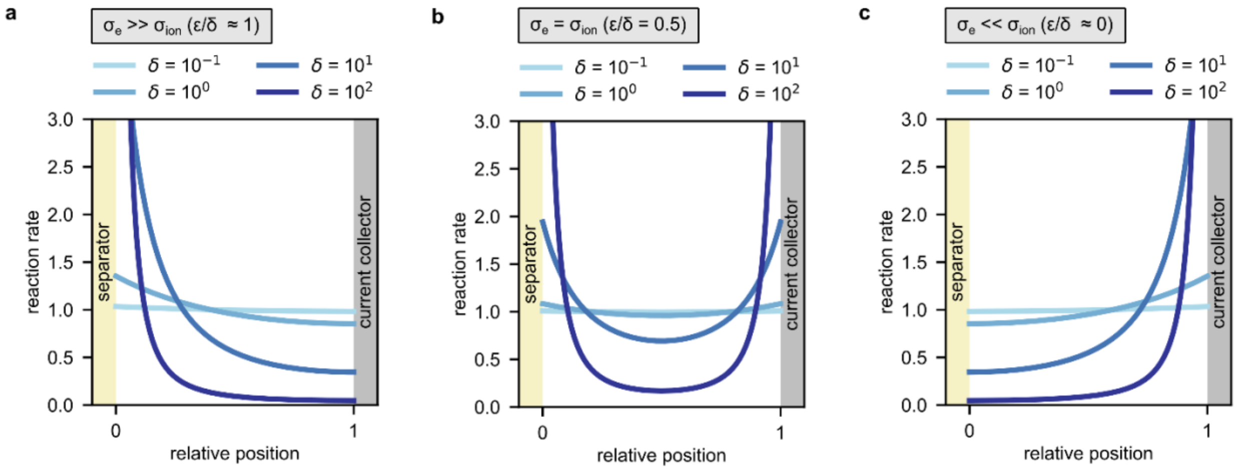

Figure S1: Reaction rate distributions of theoretical electrodes. *Distributions are calculated with δ-parameters ranging from 10^{-1} to 10^2 and **a** σ_e >> σ_{ion}, **b** $\sigma_e = \sigma_{ion}$ and **c** σ_e << σ_{ion} (adapted from Newman and Tobias[2]).*

Figure S1 shows reaction current distributions for δ-parameters ranging from 10^{-1} to 10^{2} and varying ϵ/δ. In the case of σ_{e} >> σ_{ion} (Figure S1 a), the highest reaction rates show near the separator. For equal σ_{e} and σ_{ion} (Figure S1 b), the largest rates show equally near both the separator and the current collector interface. Lastly, for σ_{e} << σ_{ion} (Figure S1 c), the highest reaction rates show close to the current collector interface.

1.4 Balancing of transport properties

Effective conductivities in a composite are governed by the conductivities of the individual phases, their volume fractions in the composite and the overall microstructure. Since the δ-parameter of an electrode is strongly influenced by the effective conductivities, factors influencing the effective conductivities directly impact δ. Hence, these factors can also be used as dials to optimize the electrode, since low δ-parameters correlate with high active material utilization (Figure 1). In the following, the composition dependency of δ is explored. For a simple example, a binary electrode composite consisting of a purely electron conducting material with conductivity $\sigma_{\mathrm{e,100\%}}$ and a purely ion conducting material with conductivity $\sigma_{\mathrm{ion,100\%}}$ is assumed. While the electron conducting phase occupies a volume fraction of φ_{e}, the ion conducting material fills up the remaining volume fraction ($\varphi_{\mathrm{ion}} = 1 - \varphi_{\mathrm{e}}$). Estimating the composition dependency of effective transport using the Bruggeman relation for spherical particles ($\sigma_{\mathrm{eff}} = \sigma_{100\%} \cdot \varphi^{3/2}$),[3] the conductivity dependent term of δ can be expressed as

$$\delta \propto \left(\frac{1}{\sigma_{\mathrm{e,eff}}} + \frac{1}{\sigma_{\mathrm{ion,eff}}}\right) \approx \left(\frac{1}{\sigma_{\mathrm{e,100\%}} \cdot {\varphi_{\mathrm{e}}}^{3/2}} + \frac{1}{\sigma_{\mathrm{ion,100\%}} \cdot (1-\varphi_{\mathrm{e}})^{3/2}}\right) \tag{S4}$$

Figure S2 shows the conductivity dependent term of δ as a function of composition, calculated using Eq. S4. While the conductivity of the purely electron-conducting phase is fixed at 10^{0} mS·cm^{-1}, that of the purely ion-conducting phase is varied between 10^{-2} mS·cm^{-1} and 10^{2} mS·cm^{-1}.

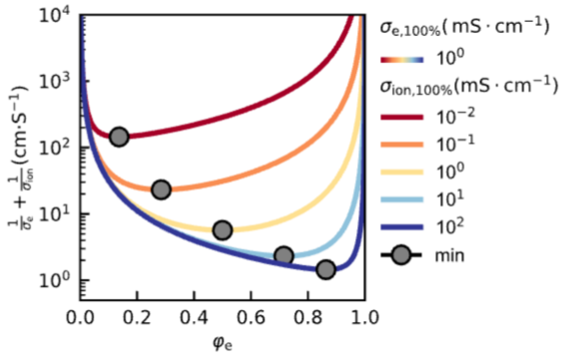

Figure S2: Conductivity dependent term of δ. *Approximated conductivity dependent term of δ as a function of volume fraction of the electron conducting phase (φ_e). The conductivity of the purely electron-conducting phase ($\sigma_{e,100\%}$) is fixed at 10^{0} mS·cm^{-1}. The conductivity of the purely ion-conducting ($\sigma_{ion,100\%}$) phase is varied between 10^{-2} mS·cm^{-1} and 10^{2} mS·cm^{-1}. Each minimum conductivity dependent term per material system is marked with a circle.*

The conductivity term is small when the individual components show high ionic and electronic conductivities. The minimum of the curves corresponds to the optimal composition (in terms of low δ) for the theoretical composites explored in this example. This minimum is highly dependent on the conductivities of the individual components: When $\sigma_{\mathrm{e,100\%}}$ = $\sigma_{\mathrm{ion,100\%}}$, equal volume fractions of ion and electron conducting phase yield the lowest conductivity dependent term. A shift towards lower or higher φ_{e}, is observed however, when $\sigma_{\mathrm{e,100\%}}$ > $\sigma_{\mathrm{ion,100\%}}$ or $\sigma_{\mathrm{e,100\%}}$ < $\sigma_{\mathrm{ion,100\%}}$ are chosen respectively. This example highlights, that balancing conductivities to minimize the conductivity dependent term can be a helpful strategy to support design towards small δ-parameters. This implies that the ideal volumetric ratios of active material and solid electrolyte strongly depend on the conductivities of the pure components of the composite electrode. Minimization of the conductivity dependent term of δ enables to gauge the range in which an optimized reaction current distribution can be expected. This can be used as basis for experimental planning to more efficiently achieve ideal electrode design for composite electrodes with high loadings and charging rates.

S2 Experimental Section

If not stated otherwise, all preparative work was conducted in a glovebox under argon atmosphere (H_2O < 0.5 ppm, O_2 < 0.5 ppm).

Synthesis: For the syntheses of Li_6PS_5Cl and $Li_{5.5}PS_{4.5}Cl_{1.5}$, lithium chloride (LiCl, Alfa-Aesar, 99%), lithium sulfide (Li_2S, Alfa-Aesar, 99.9%) and phosphorus pentasulfide (P_4S_{10}, Merck, 99%) were mixed in stochiometric ratio and manually ground in an agate mortar for 15 minutes. The obtained powder was pressed into pellets and then filled into carbon coated quartz ampules with an approximate length of 10 cm to 12 cm and a diameter of 10 mm, which were previously dried at 800 °C under dynamic vacuum (p < 10 mbar) for 2 h. The ampules were sealed under vacuum and subsequently transferred into a tube furnace. Both SE were heated using a heating rate of 100 $°C \cdot h^{-1}$. While Li_6PS_5Cl was heated to 550 °C for 14 days, $Li_{5.5}PS_{4.5}Cl_{1.5}$ was synthesized via a two-step route, where the pellets were first kept at 450 °C for 3 days, then thoroughly ground, pressed into pellets and heated for another 3 days at 450 °C. The obtained pellets were hand ground for further characterization and stored in a glovebox. Subsequently, their phase purity was confirmed by powder X-ray diffraction and their ionic conductivity and particle size distribution were determined (Figure S3).

Powder X-ray diffraction: All samples were characterized in sealed borosilicate glass capillaries (Hilgenberg, 0.5 mm diameter). The measurements were conducted in Debye-Scherrer geometry at 298 K using a STOE Stadi P diffractometer. Mo-K_α radiation with a wavelength of 0.7093 Å, two Mythen 2k detectors and a curved Ge(111) monochrometer were employed. Diffraction experiments were performed in a Q-range of 0.62 $Å^{-1}$ to 8.17 $Å^{-1}$, using a 2 θ step size of 0.1° and measuring for 20 s per step.

Potentiostatic electrochemical impedance spectroscopy (PEIS): To determine the ionic conductivity of the SE by AC impedance measurements, approximately 100 mg of as-synthesized powders were filled into a press cell. Subsequently, the SE was densified by applying 374 MPa for 3 min. PEIS was measured by applying an excitation amplitude of 10 mV in a frequency range of 7 MHz to 1 Hz.

Assembly of solid-state batteries: Half-cells with the configuration In/InLi|SE|SE-NCM83 were assembled in an airtight press cell under argon atmosphere. In the first step, 80 mg NCM83 (NCM83, MSE supplies, dried overnight under dynamic vacuum at 250 °C) was mixed with 20 mg catholyte (corresponding to a volume ratio of 58:42) using a FRITSCH P-23 vibratory ball mill at 15 Hz for 15 min with 5 ZrO_2 milling media (Ø = 5 mm). Subsequently, 80 mg SE were added as a separator and hand pressed. Next, the cathode composite was added (24.6 mg for 5.01 $mAh \cdot cm^{-2}$, 29.5 mg for 6.01 $mAh \cdot cm^{-2}$ and 34.4 mg for 7.01 $mAh \cdot cm^{-2}$), evenly distributed across the surface of the separator and densified by applying 374 MPa for 3 min on the cell stack. Then, In foil (chemPUR, 99.999%, 100 µm, Ø = 9 mm) was added on the other side of the separator. To prepare the In/InLi negative electrode, pieces of a Li rod (abcr, 99.8%) with an approximate mass of 0.8 mg were pressed into a Li foil and stacked on top of the In layer to form a In/InLi alloy *in situ*. Finally, all press cells were closed and fixed in a metal frame at 60 MPa stack pressure.

For the *operando* HEXRD a special custom-made cell housing was used.[4] In the first step, the solid electrolyte (100 mg cm-2) was filled into the cell and compressed to separator layer. The respective cathode composite with an areal loading of 7 mAh cm^{-2} was uniformly distributed on the separator. The two layers were densified with 50 MPa for 3 min in an uniaxial press. The In foil with a diameter of 3.5 mm was placed on the other side of the separator. The Li foil (0.2 mg), prepared as described above, was placed on top of the In foil. For cycling a stack pressure of 60 MPa was applied.

Electrochemical testing: After resting all half-cells at OCV for 6h, two different electrochemical experiments were conducted. To test continuous cycling in a potential window of 2.0 V to 3.7 V vs. In/InLi (2.62 V to 4.32 V vs. Li^+/Li), the In/InLi|SE|SE-NCM83 half-cells were cycled for 50 cycles at 298 K, using a C-rate of 0.1 C based on 200 $mAh \cdot g_{CAM}^{-1}$ theoretical capacity of NCM83. This corresponds to current densities of 0.501 $mA \cdot cm^{-2}$, 0.601 $mA \cdot cm^{-2}$ and 0.701 $mA \cdot cm^{-2}$ for the areal active material loadings of 5.01 $mAh \cdot cm^{-2}$, 6.01 $mAh \cdot cm^{-2}$ and 7.01 $mAh \cdot cm^{-2}$, respectively. For each testing condition, three cells were built. Additionally, C-rate tests were conducted on half-cells. The charge and discharge currents in the voltage window from 2.0 V to 3.7 V vs. In/InLi for CAM loadings of 5.01 $mAh \cdot cm^{-2}$ and 7.01 $mAh \cdot cm^{-2}$ are given in Table S1.

Table S1: *Current densities employed for the respective CAM loadings at each C-rate.*

C-rate	*j* of 5.01 mAh·cm^{-2} cells (mA·cm^{-2})	*j* of 7.01 mAh·cm^{-2} cells (mA·cm^{-2})
**C/40**	0.125	0.175
**C/20**	0.251	0.351
**C/10**	0.501	0.701
**C/5**	1.003	1.402
**C/3**	1.671	2.337

Effective conductivity characterization: Symmetrical cells were assembled. To determine the effective ionic conductivities, 100 mg of cathode composite was inserted into press cells and was pressed uniaxially at a pressure of 374 MPa for 3 min. Subsequently, an electron blocking Li_6PS_5Cl layer was added on either side and pressed at 374 MPa for 3 min again. Next, In foil (chemPUR, 99.999%, 100 µm, Ø = 9 mm) and freshly prepared Li foil (Li, abcr, 99.8%, 1.5 mg) were added on either side and the cells were fixed into an aluminum frame, keeping the cell at 60 MPa stack pressure. To determine the effective electronic conductivities, 100 mg of cathode composite was inserted into press cells and uniaxially pressed at 374 MPa for 3 min and the cell was subsequently fixed into an aluminum frame to keep it at 60 MPa stack pressure. After a 6 h equilibration period at 25 °C, PEIS was measured on all symmetric cells using a perturbation amplitude of 10 mV in a frequency range of 10 mHz to 7 MHz. All spectra were analyzed with RelaxIS 3 applying the T-type transition line model (TLM). Subsequently, DC polarization was performed to corroborate the TLM results. For DC polarization experiments on the cells for effective ionic conductivity determination, voltage steps from 0.5 mV to 5 mV with a step size of 0.5 mV were conducted applying each voltage for 5 h to ensure steady-state conditions. The DC polarization experiments on the effective electronic conductivity cells was performed by applying voltage steps in a range from 5 mV to 50 mV with a step size of 5 mV, keeping each voltage for 2 h.

***Operando* high energy X-ray diffraction:** The electrochemical cycling during HEXRD was performed at room temperature using a PalmSens4. The cells were cycled with 0.7 mA cm-2 (0.1 C) in a voltage range from 2.0 – 3.7 V vs In/LiIn. Spatially-scanning HEXRD experiments were performed in transmission geometry at the P07B beamline (PETRA III synchrotron, DESY, Hamburg) using a 4 mm diameter cell optimized for operando solid-state battery measurements (Figure S5). The photon energy was set to 73.3 keV, and the beam size was set to 1 × 0.01 mm (vertical × horizontal). The sample was scanned across the incoming beam with a step size of 0.01 mm, acquiring a diffraction pattern at each point using a PerkinElmer XRD 1621 FlatPanel X-ray detector with an acquisition time of 2 s per frame. The sample-to-detector distance was calibrated to 1.481 mm using a LaB6 standard.

Analysis of the particle size distribution: To determine the volume-weighted particle size distribution a HELOS particle size analyzer by Sympatec was used. 2.5 mg of SE was placed in a vial and dispersed in 2 ml of *p*-xylene mixed with 1 wt.-% polyisobutene to enhance the viscosity. Subsequently, the mixture was ultrasonicated with an ultrasonic finger for 15 min in glovebox atmosphere. Next, the dispersion was added dropwise into a cuvette filled with 40 ml of *p*-xylene with 1 wt.-% polyisobutene outside the glovebox. The dispersion was continuously stirred to prevent sedimentation of particles during the measurement by laser diffraction.

Resistor network simulations: Effective conductivities of NCM-Li_6PS_5Cl and NCM- $Li_{5.5}PS_{4.5}Cl_{1.5}$ have been estimated using a previously reported resistor network model.[5] The NCM phase has been assumed to be exclusively electron conducting and the solid electrolyte phases were assumed to be exclusively ion conducting. A total of 100x100x100 voxels was used to build the virtual microstructures. The number of voxels corresponding to the active material and solid electrolyte phase are determined by the specified solid electrolyte volume fractions (20 %, 30 %, 40 %, 50 %, 60 %, 70 %, 80 %). To account for larger solid electrolyte domains embedded in a continuous matrix of smaller NCM particles, clusters of 500 voxels were iteratively inserted into a continuous matrix describing the NCM phase when building the virtual microstructures.

S3 Characterization of solid electrolytes and composite cathodes

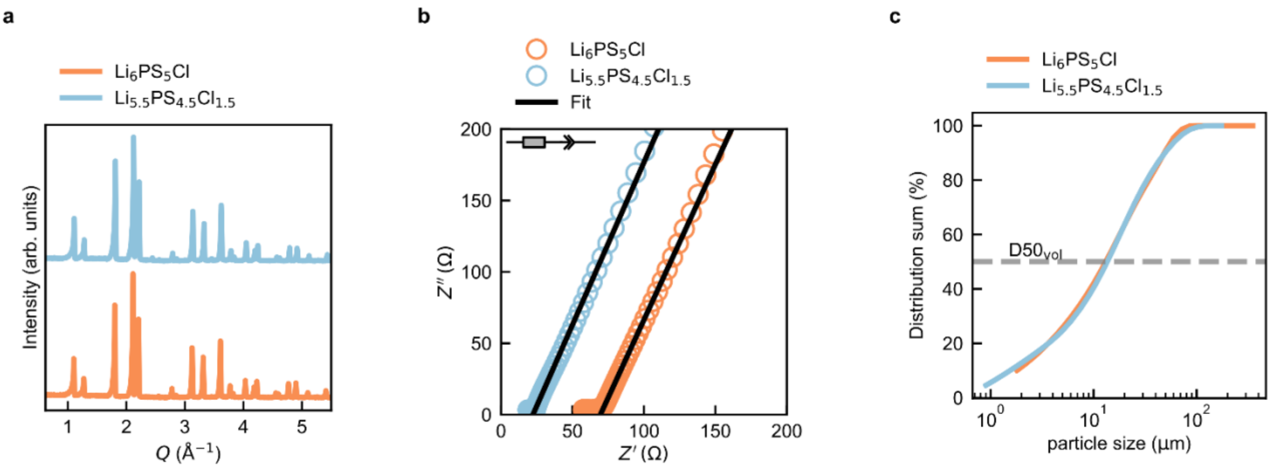

Figure S3*: *Characterization of the solid electrolytes. *Data corresponding to Li_6PS_5Cl and $Li_{5.5}PS_{4.5}Cl_{1.5}$ are shown in orange and blue respectively.* ***a*** *Powder X-ray diffraction patterns* ***b*** *Impedance spectra and corresponding fit with a resistor and CPE connected in series* ***c*** *Particle size distributions measured by Laser diffraction.*

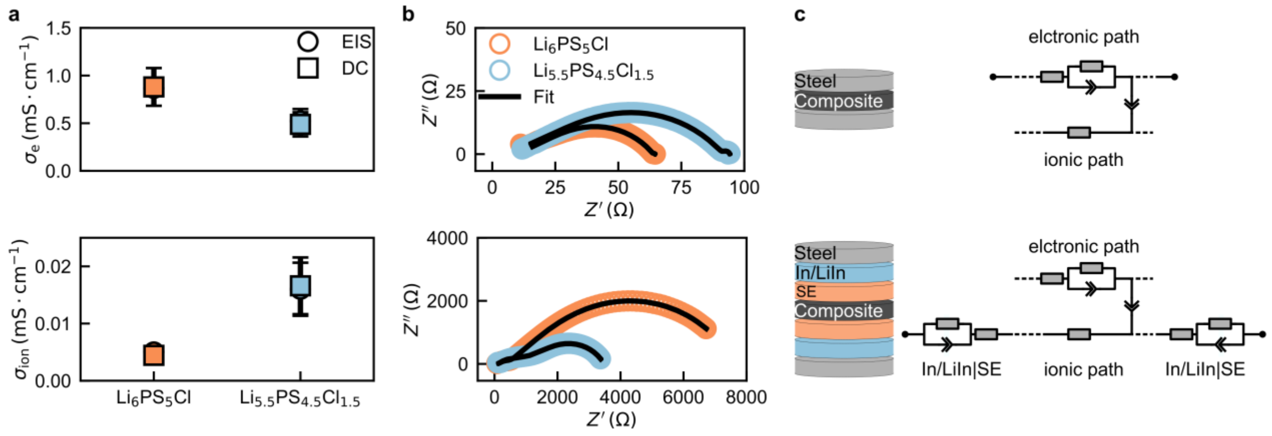

Figure S4*: *Determining the partial conductivities of NCM83-Argyrodite composite electrodes *Data of composites utilizing Li_6PS_5Cl and $Li_{5.5}PS_{4.5}Cl_{1.5}$ as catholytes are shown in orange and blue respectively* ***a*** *Partial ionic and electronic conductivities determined from triplicate measurements of DC polarization and impedance spectroscopy measurements. The error bars correspond to the standard deviation.* ***b*** *Impedance spectra and corresponding fits with a TLM model.* ***c*** *Experimental setups used to conduct impedance spectroscopy and DC polarization experiments as well as equivalent circuits of the TLM models used to evaluate the impedance results.*

S4 Operando high-energy X-ray diffraction

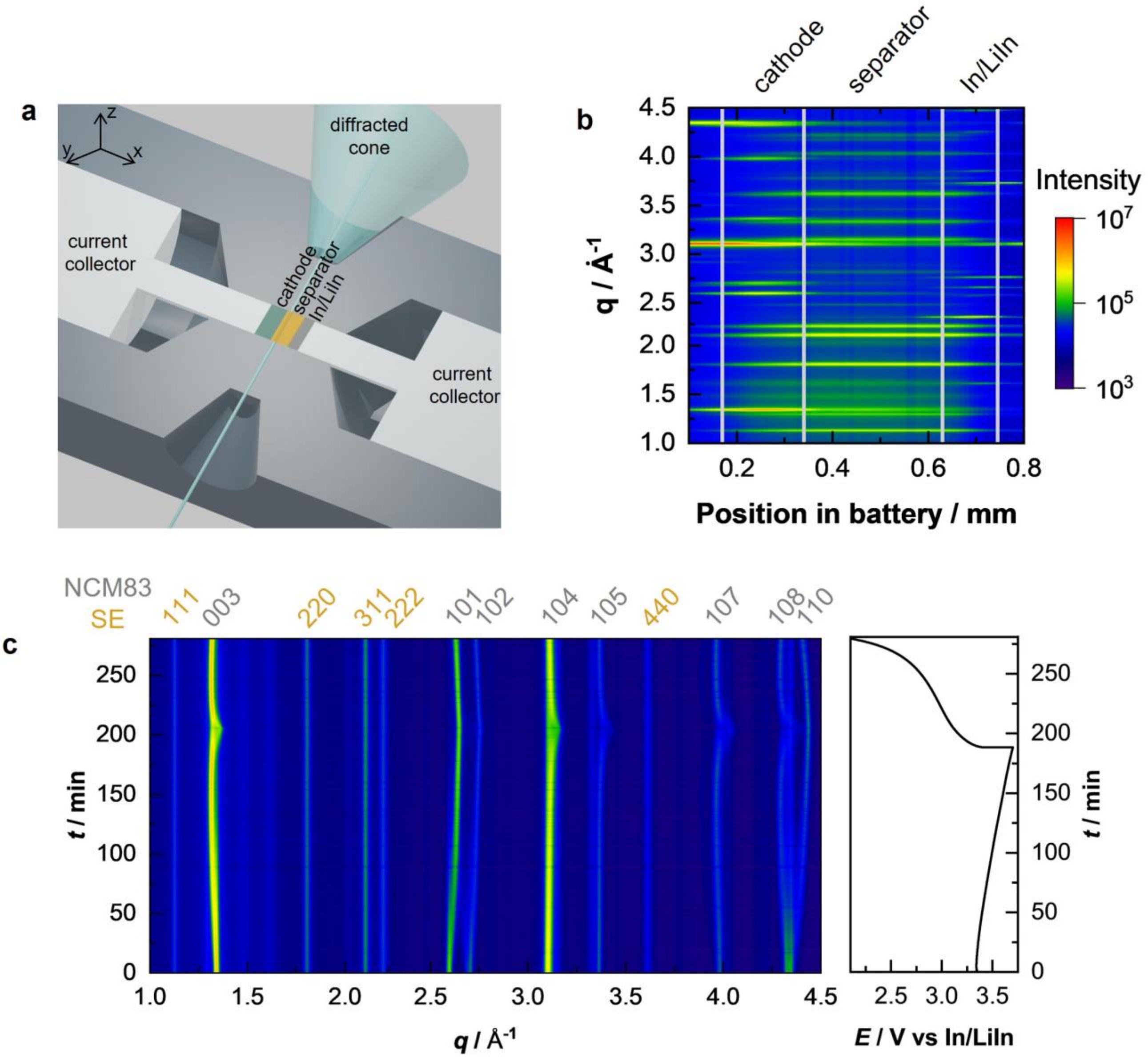

Figure S5: Principles of HEXRD. *a) Schematic cell setup with cell housing made of PEEK and supporting brass shell. A window in the brass shell and a reduced PEEK wall thickness serve as the measuring point. The cell case is sealed airtight with O-rings and stack pressure is applied by a frame. The measurement is taken along the x-axis. b) Complete scan of NCM83-Li_6PS_5Cl | $Li_{5.5}PS_{4.5}Cl_{1.5}$ | In/LiIn cell before cycling to locate the positions of the cathode, separator and In/LiIn electrode. c) Change in HEXRD pattern of cathode composite at one position in cathode during charge and discharge over time. Miller indices of visible reflections for NCM83 in gray and of Li_6PS_5Cl in yellow.*

4.1 Reaction distribution

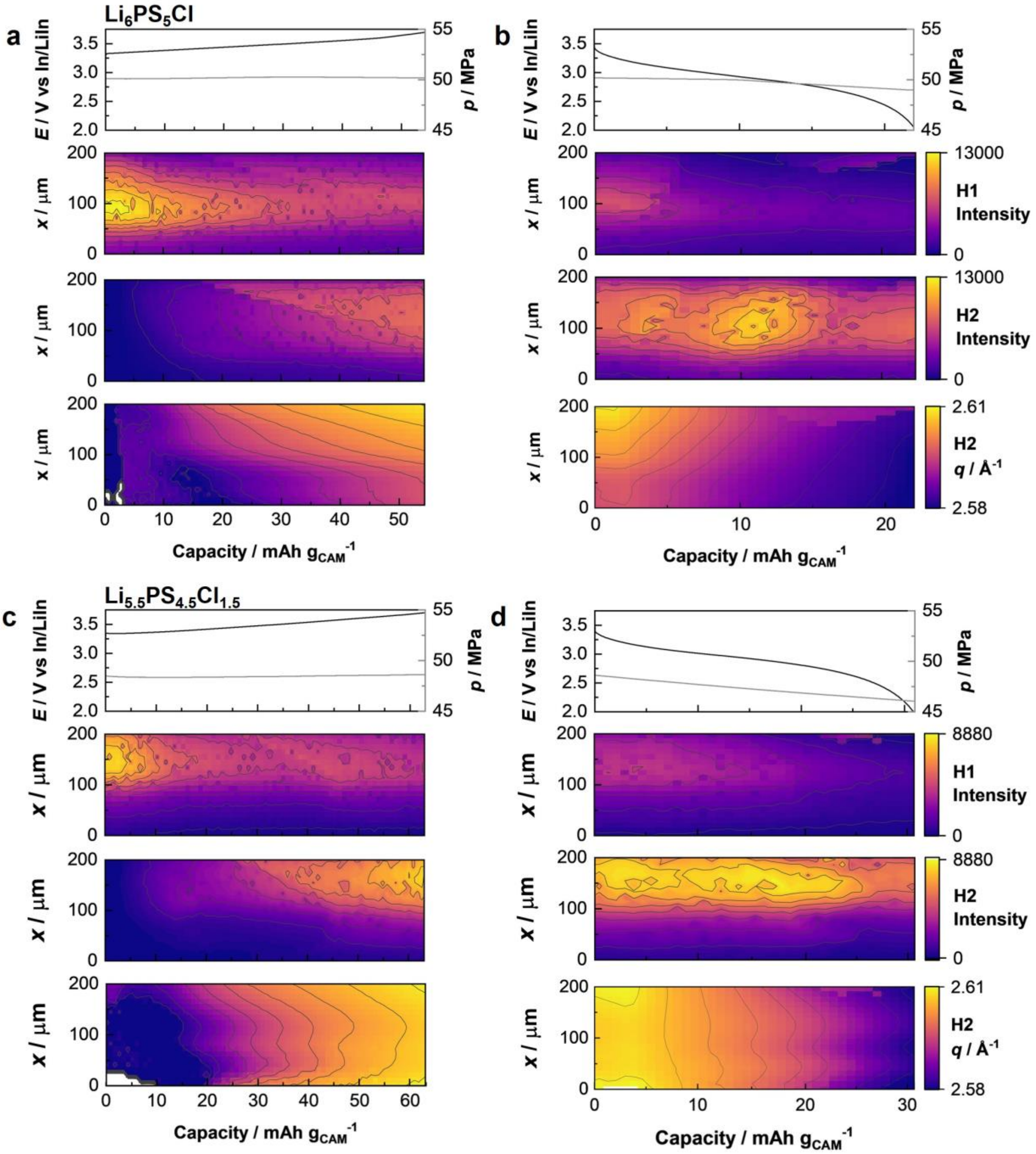

Figure S6: Results of operando HEXRD. *Voltage change, intensity of (101) reflection of H1 and H2 phase and displacement of (101) reflection according to capacity during charge (a & c) and discharge (b & d) of cathode composites with Li_6PS_5Cl (a & b) and $Li_{5.5}PS_{4.5}Cl_{1.5}$ (c & d). The contour plots of the intensities of (101) reflection of H1 and H2 phase show that during delithiation (a & c), the intensity of the reflection of the H1 decreases while the intensity of the H2 phase increases. This is consistent with literature on the different phases of NCM during delithiation.[6,7] However, during lithiation (b & d) H1 phase does not reform, which can explain the charge losses in the first cycle.*

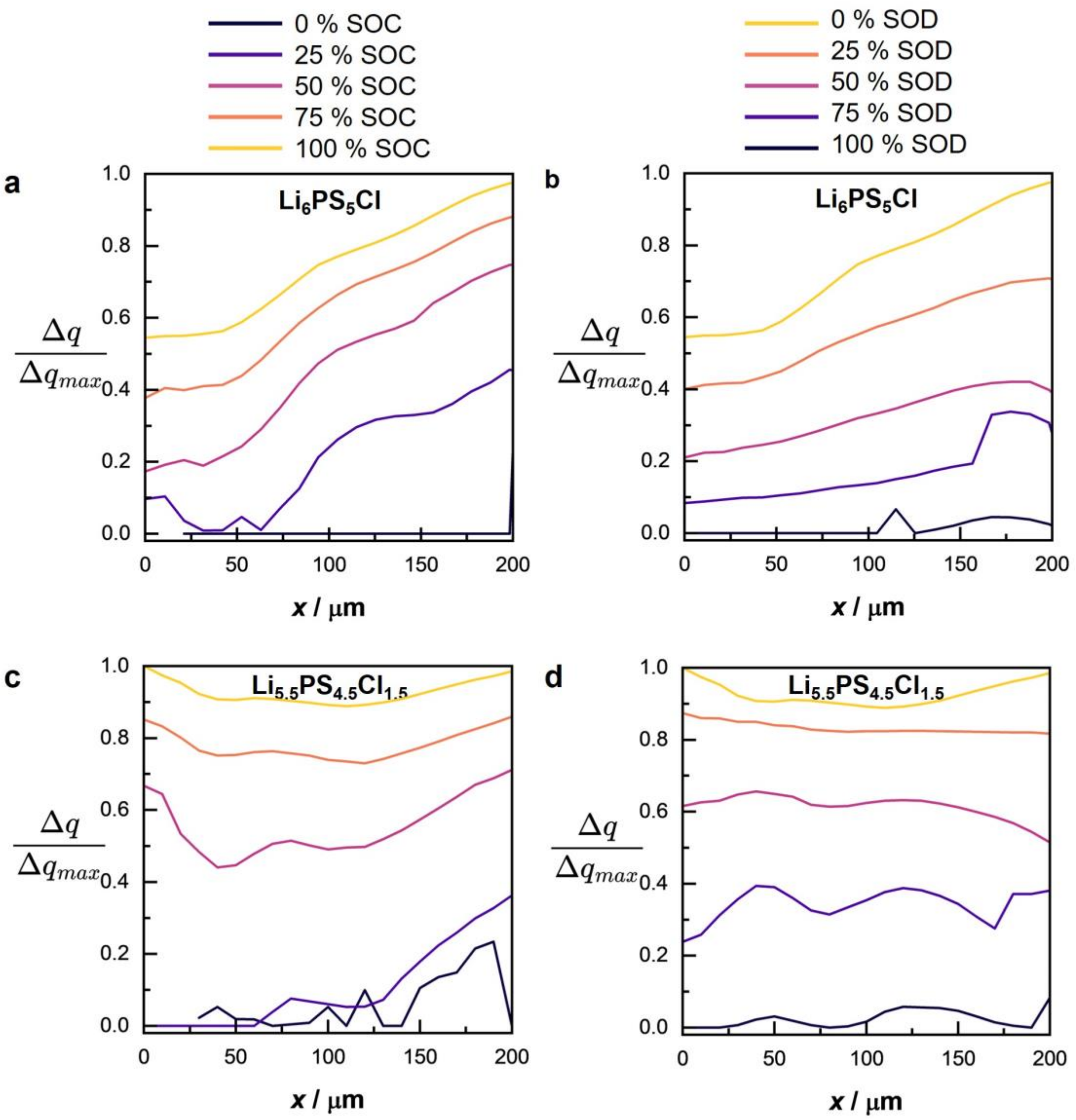

Figure S7: Normalized displacement of (101) reflection*. The displacement of (101) reflection Δq is normalized to the maximum displacement Δq_{max} over the thickness of cathode x for different state of charge (SOC, a &c) or discharge (SOD, b & d). Composite cathode with Li_6PS_5Cl (a) shows significant gradient in the displacement over the thickness of cathode during charge. At separator side of cathode (x = 200 µm), a higher shift is visible while half of the maximum shift was achieved on the current collector side (x = 0 µm). This indicates a strong gradient in delithiation. At the beginning of discharge (b) the gradient in shift is visible. However, at the end of discharge the (101) diffraction peak shifted back to pristine position. The composite cathode with $Li_{5.5}PS_{4.5}Cl_{1.5}$ shows similar behavior as composite with Li_6PS_5Cl regarding a higher displacement of (101) reflection close to the separator compared to the current collector side while charge (c) and discharge (d). However, the gradient in displacement is less pronounced for the cell with $Li_{5.5}PS_{4.5}Cl_{1.5}$.*

4.2 Strain evolution of solid electrolyte

To investigate the lattice strain $\varepsilon = \frac{\Delta d}{d}$ of the solid electrolyte within the composite cathode that occurs during cycling, the distortion of (440) reflection of the solid electrolyte is analyzed (Figure S5). The obtained HEXRD patterns were azimuthally integrated in 4° circular sections across the full range (0 to 2θ). Subsequently, the diffraction peaks for each azimuthal section were fitted using a Lorentz function. Strain evaluation $\varepsilon = \frac{\Delta d}{d}$, was then performed by applying the modified sin²y method.[8] The azimuthal integration itself was performed using the MatFRAIA package in MATLAB.[9]

For strain evaluation, maximizing precision requires utilizing reflection with a large scattering *q* vector, which corresponds to higher diffraction angles. The (440) reflection is therefore selected as it provides the optimal balance between a high *q* value and sufficient diffraction intensity for a reliable analysis. The solid electrolyte in the composite electrode serves as the best candidate for analyzing the chemomechanical strain because it does not exhibit any phase changes during battery cycling and is directly affected by the contact to the expanding and contracting active material. Furthermore, due to the small Young modulus of the argyrodites (*E* = 22 GPa)[10] compared to the modulus of NCM-materials (*E* = 150 GPa)[11], any volume change in the cell likely results directly in strain in the solid electrolyte.

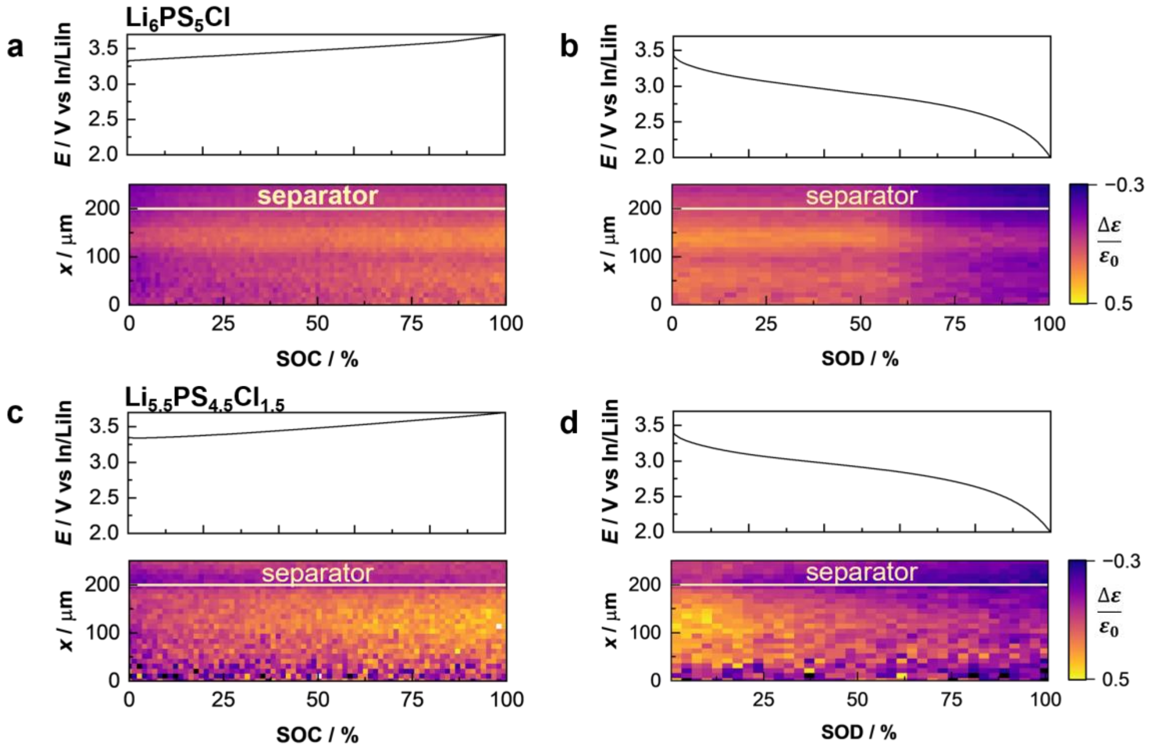

Figure S8: **Strain evolution of argyrodite during cycling**. *Voltage change and the change of solid electrolyte strain* $\Delta\varepsilon$ *normalized to the pristine state of strain* ε_0 *as a function of the state of charge (a & c) and discharge (b & d) of cathode composites with* Li_6PS_5Cl *(a & b) and* $Li_{5.5}PS_{4.5}Cl_{1.5}$ *(c & d). Approximate transition from cathode to separator is marked with a yellow line. Positive value for* $\frac{\Delta\varepsilon}{\varepsilon_0}$ *indicates an increase in compressive strain while a negative value indicates less compressive strain compared to pristine state.*

During charge the evolution of a compressive strain on the solid electrolyte is visible. The compressive strain in the solid electrolyte can be explained by a volume increase of the In/LiIn electrode of ~106 %, which has been shown before to lead to densification effects.[12,13] However, Figure S8 shows a higher change in strain in the electrodes compared to the separator. This indicates that the compressive strain cannot be explained by the volume expansion of the In/LiIn electrode alone. An additional contribution to local strain in the solid electrolyte comes from volume changes of NCM83. The volume change of NCM83 is anisotropic resulting in an expansion along the *c*-direction.[14]

The strain for the cathode with Li_6PS_5Cl (Figure S8a) shows a change of strain at the beginning of delithiation close to the separator. With further delithiation the change of strain becomes visible on the side of the current collector. Therefore, a strain alteration gradient can be seen along the length of the cathode, in line with the reaction front observed and lithiation gradient (Figure S6). During discharge (Figure S8b), the strain returns to its initial position shown by $\frac{\Delta\varepsilon}{\varepsilon_0}$

close to 0. The change of strain of $Li_{5.5}PS_{4.5}Cl_{1.5}$ (Figure S8c) begins close to the separator side and progresses toward the current collector with further delithiation. The strain returns to the pristine state during discharge (Figure S8d). While the gradient is more pronounced in the cell using Li_6PS_5Cl as the catholyte, stronger changes of strain are visible for $Li_{5.5}PS_{4.5}Cl_{1.5}$, potentially stemming from more active material being addressed electrochemically. The return to the pristine state of strain in Li_6PS_5Cl and $Li_{5.5}PS_{4.5}Cl_{1.5}$ indicates a good adaptation to volume changes during delithiation and lithiation of NCM83. This is consistent with previous studies on the mechanical properties of sulfide solid electrolytes.[12,15,16] However, the clear non-uniform change of strain indicates a non-uniform volume change over the electrode. To relieve the resulting stress new fractures and interfacial cracks may form[12,17], and the formation of these cracks and fractures results in contact loss which is a major factor in capacity fading.[18] While these cracks cannot be resolved here, any contact loss leads to inactive NCM83, resulting in longer diffusion paths for Li^+-ions, which in turn exacerbates existing non-uniform reaction distributions. Nevertheless, sulfide electrolytes exhibit reversible strain, enabling them to adapt well to volume changes at the particle level.

References

[1] J. Janek, W. G. Zeier, “Challenges in speeding up solid-state battery development” *Nat. Energy* **2023**, *8*, 230–240.

[2] J. S. Newman, C. W. Tobias, “Theoretical analysis of current distribution in porous electrodes” *J. Electrochem. Soc.* **1962**, *109*, 1183.

[3] B. Tjaden, S. J. Cooper, D. J. L. Brett, D. Kramer, P. R. Shearing, “On the origin and application of the Bruggeman correlation for analysing transport phenomena in electrochemical systems” *Curr. Opin. Chem. Eng.* **2016**, *12*, 44–51.

[4] S. Mičky, E. Šimon, J. Todt, K. Végsö, P. Nádaždy, P. Krížik, E. Majková, J. Keckes, J. Li, P. Siffalovic, “Operando Spatial and Temporal Tracking of Axial Stresses and Interfaces in Solid-state Batteries” *Small* **2024**, *20*, 2307837.

[5] L. Ketter, N. Greb, T. Bernges, W. G. Zeier, “Using resistor network models to predict the transport properties of solid-state battery composites” *Nat. Commun.* **2025**, *16*, 1–9.

[6] L. Yu, J. Wang, T. Zhou, W. Huang, T. Li, L. Ma, X. Xiao, S.-B. Son, S. N. Ehrlich, J. Wen, “Unraveling the origin of air-stability in single-crystalline layered oxide positive electrode materials” *Nat. Commun.* **2025**, *16*, 6519.

[7] I. Buchberger, S. Seidlmayer, A. Pokharel, M. Piana, J. Hattendorff, P. Kudejova, R. Gilles, H. A. Gasteiger, “Aging analysis of graphite/$LiNi_{1/3}Mn_{1/3}Co_{1/3}O_2$ cells using XRD, PGAA, and AC impedance” *J. Electrochem. Soc.* **2015**, *162*, A2737.

[8] M. Stefenelli, J. Todt, A. Riedl, W. Ecker, T. Müller, R. Daniel, M. Burghammer, J. Keckes, “X-ray analysis of residual stress gradients in TiN coatings by a Laplace space approach and cross-sectional nanodiffraction: a critical comparison” *Applied Crystallography* **2013**, *46*, 1378–1385.

[9] A. B. Jensen, T. E. K. Christensen, C. Weninger, H. Birkedal, “Very large-scale diffraction investigations enabled by a matrix-multiplication facilitated radial and azimuthal integration algorithm: MatFRAIA” *Synchrotron Radiation* **2022**, *29*, 1420–1428.

[10] Z. Deng, Z. Wang, I.-H. Chu, J. Luo, S. P. Ong, “Elastic properties of alkali superionic conductor electrolytes from first principles calculations” *J. Electrochem. Soc.* **2015**, *163*, A67.

[11] D. Kim, H. C. Shim, T. G. Yun, S. Hyun, S. M. Han, “High throughput combinatorial analysis of mechanical and electrochemical properties of $Li[Ni_xCo_yMn_z]O_2$ cathode” *Extreme Mech. Lett.* **2016**, *9*, 439–448.

[12] W. Zhang, D. Schröder, T. Arlt, I. Manke, R. Koerver, R. Pinedo, D. A. Weber, J. Sann, W. G. Zeier, J. Janek, “(Electro) chemical expansion during cycling: monitoring the pressure changes in operating solid-state lithium batteries” *J. Mater. Chem. A Mater.* **2017**, *5*, 9929–9936.

[13] C. D. Alt, S. Keuntje, I. L. Schneider, J. Westphal, P. Minnmann, J. K. Eckhardt, K. Peppler, J. Janek, “In–Li Counter Electrodes in Solid-State Batteries–A Comparative Approach on Kinetics, Microstructure, and Chemomechanics” *Adv. Energy Mater.* **2025**, *15*, 2404055.

[14] A. O. Kondrakov, A. Schmidt, J. Xu, H. Geßwein, R. Mönig, P. Hartmann, H. Sommer, T. Brezesinski, J. Janek, “Anisotropic lattice strain and mechanical degradation of high-and low-nickel NCM cathode materials for Li-ion batteries” *J. Phys. Chem. C* **2017**, *121*, 3286–3294.

[15] A. Sakuda, A. Hayashi, M. Tatsumisago, “Sulfide solid electrolyte with favorable mechanical property for all-solid-state lithium battery” *Sci. Rep.* **2013**, *3*, 2261.

[16] P. Perrenot, P. Bayle-Guillemaud, C. Villevieille, “Composite electrode ($LiNi_{0.6}Mn_{0.2}Co_{0.2}O_2$) engineering for thiophosphate solid-state batteries: morphological evolution and electrochemical properties” *ACS Energy Lett.* **2023**, *8*, 4957–4965.

[17] S. Kalnaus, N. J. Dudney, A. S. Westover, E. Herbert, S. Hackney, “Solid-state batteries: The critical role of mechanics” *Science* **2023**, *381*, eabg5998.

[18] R. Koerver, I. Aygün, T. Leichtweiß, C. Dietrich, W. Zhang, J. O. Binder, P. Hartmann, W. G. Zeier, J. Janek, “Capacity fade in solid-state batteries: interphase formation and chemomechanical processes in nickel-rich layered oxide cathodes and lithium thiophosphate solid electrolytes” *Chem. Mater.* **2017**, *29*, 5574–5582.
